# Renal Function in Children Suffering from Sickle Cell Disease: Challenge of Early Detection in Highly Resource-Scarce Settings

**DOI:** 10.1371/journal.pone.0096561

**Published:** 2014-05-08

**Authors:** Michel Ntetani Aloni, René Makwala Ngiyulu, Jean-Lambert Gini-Ehungu, Célestin Ndosimao Nsibu, Mathilde Bothale Ekila, François Bompeka Lepira, Nazaire Mangani Nseka

**Affiliations:** 1 Division of Hemato-oncology and Nephrology, Department of Pediatrics, University Hospital of Kinshasa, School of Medicine, University of Kinshasa, Kinshasa, Democratic Republic of Congo; 2 Intensive Care Division, Department of Pediatrics, University Hospital of Kinshasa, School of Medicine, University of Kinshasa, Kinshasa, Democratic Republic of Congo; 3 Department of Internal Medicine, University Hospital of Kinshasa, School of Medicine, University of Kinshasa, Kinshasa, Democratic Republic of Congo; 4 Division of Nephrology and Dialysis, Department of Internal Medicine, University Hospital of Kinshasa, School of Medicine, University of Kinshasa, Kinshasa, Democratic Republic of Congo; Southern Illinois University School of Medicine, United States of America

## Abstract

**Background:**

The prevalence of Sickle cell disease is extremely high in Democratic Republic of Congo. Despite this high prevalence of the disease, data on renal abnormalities in children are rare.

**Method:**

The study proposed to assess blood pressure, glomerular function, urea and uric acid levels in 65 steady state Congolese children with homozygous sickle cell disease and 67 normal controls.

**Results:**

In Hb-SS group, blood pressure level tended to be lower than Hb-AA groups but there was no statistically significant difference (p>0.05) between the two groups. The absolute values for GFR corrected for BSA were significantly higher in Hb-SS group compared to Hb-AA group (130.5±34.1 ml/min/1.73 m^2^ vs 113.7±24.5 ml/min/1.73 m^2^; p = 0.004). Children with Hb-SS were more likely to hyperfiltrate (30.8% of subjects) than children with Hb-AA (6.1% of subjects). Proteinuria was found in 4 (6.2%) children with Hb-SS. Uric acid level was significantly increased in children with Hb-SS compared to corresponding values in control group (4.4±1.3 mg/dl vs 3.5±1.1 mg/dl; p<0.001). Urea level was significantly decreased compared to corresponding values in Hb-AA group (15.3±8.3 mg/dl vs 22.9±10.1 mg/dl; p<0.001).

**Conclusion:**

Hyperfiltration, low creatinine, lower urea and high uric acid are more common in children with sickle cell disease than in normal controls.

## Introduction

Sickle cell disease (SCD) is an autosomal recessive genetic condition due to a mutation in the beta-globin gene resulting in replacement of glutamic acid in position 6 of the beta-globin chain by valine resulting in an abnormal haemoglobin HbS molecule. SCD is the commonest genetic disease worldwide. The highest frequencies of homozygous SCD in the world occur in sub-Saharan Africa where 3 to 4% of populations are affected [1]. The sickle cell genes occur commonly in areas of the world with intense malaria transmission.

Democratic Republic of Congo (DRC) has the second highest population of SCD patients in the whole world after Nigeria. The prevalence of SCD is extremely high with 25 to 30% of sickle cell trait carrier in the general population. Recent population-based studies have calculated the prevalence to be 1.4% in Congolese newborns and the incidence to be approximately 50,000 newborns per year [1], [2].

The kidney is an organ of considerable impact on the clinical course of sickle cell patients. In DRC, the main haplotype of SCD is the Central African Republic (CAR) –globin gene, the most severe form of the disease. The CAR βs globin gene haplotype was found significantly more often in patients with chronic renal failure (CRF) suggesting a genetic predilection [3].

Despite this high prevalence of the disease in our midst and the risk of CRF, information about renal complications in pediatric population suffering from SCD in DRC are unknown. Probably this renal impairment is under-reported in African children, poverty and the paucity of pediatric nephrologists and hematologists in this region should contribute to this fact. In addition, SCD and renal diseases are not regarded as a major health problem in DRC confronted to infectious diseases and malnutrition [4], [5].

The objective of this survey was to investigate early detection of renal abnormalities in children suffering from SCD in a context of limited resource settings.

Our ultimate goals are to develop the basis for designing and implementing effective preventive interventions for renal complications in sickle cell patients. These researches also seek to inform clinical practice, education and counseling guidelines. In this first report, we assess glomerular function in children suffering from homozygous SCD in Kinshasa, DRC.

## Materials and Methods

### Ethical consideration

Since all participants were minors, they provided assent and their legal guardians provided consent for study participation. This consent procedure was reviewed and approved by the National Ethical Committee of the Public Health School of the University of Kinshasa, Kinshasa, DRC.

### Study design and population

The present cross-sectional study is the first part of a larger ongoing study of renal complications in Congolese sickle cell patients suffering from SCD. The study was conducted in 2 hospitals of Kinshasa. These hospitals were University Hospital of Kinshasa (Division of pediatric nephrology) and Sickle cell centre of Yolo. These hospitals provide most of the non-private paediatrics beds in Kinshasa for sickle cell patients.

Patients were selected in the outpatient clinic of the Pediatric Hematology Unit of the University Hospital of Kinshasa and in the outpatient clinic of Sickle Cell Centre of Yolo. The starting number was randomly chosen from the first three in the section call. For each case, one control child matched for age, sex and place of residence were recruited into the study.

Five ml of blood sample were collected and were screened for haemoglobin phenotypes at the Laboratory of Haematology of Centre Médical Monkole of Kinshasa.

The following clinical and laboratory information were collected and analyzed (i) Demographic characteristics (ii) blood pressure (iii) creatinine, uric acid and urea at admission and (iv) proteinuria.

### Laboratory analysis

All patients were free of pain for at least 15 days and had not been hospitalized or transfused for at least 100 days before the study. Children with prior known proteinuria, hypertension, diabetes, HIV, HCV, renal and cardiovascular diseases were excluded by appropriate clinical and laboratory investigations. Children under hydroxyurea therapy were also excluded.

Blood samples were collected in all subjects. Sickle cell screening was performed using isoelectric focusing (IEF) technique with the Multiphor II apparatus (G E Healthcare, Little Chalfont, England). The separation of different hemoglobin (F, A, S, and other types of hemoglobin) was obtained after application on thin layer home-made agarose gel containing ampholytes pH 6–8 (ref.2117–003; Pharmalyte pH 6.7–7.7; GE Healthcare).

Sixty-five children were suffering from sickle cell disease in steady state were recruited. All children were homozygous for the β-globin S gene mutation (SS disease). The mean age of this group was 7.3±3.3 years. Sixty-seven children with normal Hb (Hb-AA) comparable for age and Body Mass Index (BMI) were selected as a control group. The characteristics of the studied patients are summarized in table-1.

**Table 1:** Characteristics of the population of the study.

Variables	Hb-AA n = 67	Hb-SS n = 65	p
Age (years)	6.7±3.2*	7.3±3.3*	ns
Weight (kg)	21.3±8.4	19.2±0.6	ns
Height (cm)	114.8±21.1	112.8±18.5	ns
BMI (kg/m^2^)	15.6±1.9	14.9±2.1	ns

*Age range: 2–13 years.

Five milliliters of blood were drawn in EDTA tubes by venipuncture. The plasma separated from the blood by centrifugation was used for estimation of creatinine, uric acid and urea. No dietary restrictions were imposed. These tests were performed in the Clinical Biochemistry laboratory of the University Hospital of Kinshasa.

### Diagnosis of renal dysfunction

The diagnosis of glomerular dysfunction was defined as the presence of at least one of these following criteria: (i) the diagnosis of hyperfiltration, (ii) diagnosis of renal failure and/or (iii) presence of positive dipstick.

Blood pressure (BP) was measured in the sitting position after 5 minutes of relax and was measured twice in left arm using a calibrated sphygmomanometer for pediatric patients (WelchAllyn, Germany) at heart level. The definition of normal BP, hypertension or hypotension had been based on age- and height-specific percentiles [6].

*Normal BP* was defined as average systolic and diastolic BP below the 90^th^ percentile for age, gender, and height. *Hypertension* was defined as average systolic or average systolic or average diastolic BP greater than the 95^th^ percentile for age, gender and height. *Hypotension* was defined as BP<5^th^ percentile for age and height.

For Glomerular Filtration Rate (GFR) determination, creatinine clearance standardized to body surface area (BSA) was calculated for children (<20 years) using formula of Schwartz 1976 [7]: Creatinine clearance, ml/min/1.73 m^2^ =  *(0.55× height, cm)/(serum creatinine, mg/dl)*.

Body surface area (BSA) (m^2^) was calculated as: 0.007184× body weight (kg)^.425^× height (cm)^.725^.

*Hyperfiltration* was defined as a GFR greater than 140 ml/min/1.73 m^2^.

Children were considered to have *renal insufficiency* if their total serum creatinine concentrations were greater than upper limits of normal for age and sex established by Schwartz et *al.* [7].

All children provided a urine sample to detect proteins by urinary strips ‘(Combur 10-test)”.Children were considered to have *Proteinuria* if three consecutive urinalyses were at least 1+ positive for protein. If positive with dipstick protein of 3+ measurement of 24-hour urinary protein was obtained.

Hyperuricaemia was defined as a serum concentration as more than 6.5 mg/100 ml.

### Data management and Statistical analysis

Statistical analysis was performed using the statistics software SPSS for windows (15.0 SPSS, Chicago). Data are represented as means ±SD when the distribution was normal and median with range when the distribution was not normal. The analysis of Student's t-test was used for comparisons of means. Categorical variables were compared using Fischer's exact test. A p value <0.05 was considered significant.

## Results

### Blood pressure

In Hb-SS group, systolic and diastolic blood pressure level tended to be lower than Hb-AA groups. However, there was no statistically significant difference between the two groups (Table-2). Blood pressure according to sex was summarized in table 2. In Hb-SS group, systolic and diastolic blood pressure level tended to be lower than in Hb-AA groups in both sex. However, there was no statistically difference between the two groups by gender.

**Table 2:** Blood pressure according Hb status and sex.

Variables	Hb-AA n = 67	Hb-SS n = 65	p
Systolic BP (mm Hg)	97.3±11.9	94.9±9.7	0.43
Diastolic BP (mmHg)	55.8±8.3	53.2±7.6	0.16
**Girls**	n = 26	n = 31	
Systolic BP (mm Hg)	98.3±11.3	94.0±9.7	0.17
Diastolic BP (mmHg)	56.0±9.1	51.7±7.4	0.10
**Boys**	n = 41	n = 34	
Systolic BP (mm Hg)	95.0±12.8	95.7±9.8	0.97
Diastolic BP (mmHg)	55.5±7.3	54.9±7.7	0.90

A significantly higher proportion (34.1%) of subjects with systolic hypotension were children with Hb-SS, compared to (4.9%) in children with Hb-AA (Table-2). Diastolic blood hypotension was rarely found in our series.

Hypertension was a rare event and was found in 1.5% of children with Hb-SS and 1.5% of children with Hb-AA.

### Glomerular filtration

#### Creatinine

Creatinine tended to be significantly lower in children with Hb-SS than in Hb-AA subjects. The difference in creatinine level between the two groups was statistically significant (Table-3).

**Table 3:** Biological profile of renal function in studied population.

Variables	Hb-AA n = 67	Hb-SS n = 65	p
Creatinine (mg/L)	0.57±0.12*	0.50±0.13*	0.004
GFR(ml/min/1.73 m^2^)	113.7±24.5	130.5±34.1	0.004
Urea (mg/dl)	22.9±10.1	15.3±8.3	0.000
Uric acid (mg/dl)	3.5±1.1	4.4±1.3	0.000

*GFR: Glomerular filtration rate.

#### Creatinine clearance

GFR was markedly increased in Hb-SS group compared to Hb-AA group. The absolute values for GFR corrected for BSA were significantly higher in Hb-SS group compared to Hb-AA group (Table-3).

A significantly higher proportion (30.8%) of subjects with hyperfiltration were children with Hb-SS, compared to (6.1%) in children with Hb-AA.

Renal insufficiency (<80 ml/min/m^2^) was respectively present in 12.3% of children suffering from SCA. None of the studied children with Hb-AA had renal insufficiency.

#### Proteinuria

Proteinuria was found in 4(6.2%) children with Hb-SS. One child with Hb-SS was found with nephrotic-range proteinuria. No case of proteinuria was detected in Hb-AA group.

### Uric acid

Uric acid tended to be higher in children with Hb-SS than in Hb-AA subjects. This tendency was significantly decreased compared to corresponding values in control group (Table-3).

A significantly higher proportion (7.7%) of subjects with hyperuricemia were children with Hb-SS compared to (1.5%) in children with Hb-AA. No case of gout was found in our series.

### Urea

Urea tended to be lower in children with Hb-SS than in Hb-AA subjects. This tendency was significantly decreased compared to corresponding values in Hb-AA group.

## Discussion

The care of children with SCD in sub-Saharan Africa is compromised by resource deficiencies that range from inadequate healthcare budgets and a paucity of appropriately trained personnel, to scarce laboratory facilities.

In Hb-SS group, systolic and diastolic BP levels tended to be lower in comparison with Hb-AA group. These results are in consonance with previous studies [8]–[10].

Hypotension was more commonly present in homozygous sickle cell group and was diagnosed respectively in 34.1% of children with Hb-SS and in 4.9% in those with Hb-AA for systolic BP. This relative hypotension is similar to that reported in literature [9]–[11]. In this series, children were comparable for anthropometrics parameters and the lower BP in SCD cannot be attributable to low weight. This situation was previously described by Horni et al [10]. These results suggest that specific patho-physiological models should be defined in SCD.

Hypertension was a rare event in our series. Only one child with Hb-SS was found to have low GFR and to be hypertensive for diastolic BP. There is little data about hypertension in children suffering from SCD. In a recent study, high frequency of abnormalities in BP measurements was reported [12]. However, hypertension in SCD is difficult to classify and normal values in sickle cell patient require more attention. Elevated BP in this population is a high predictor of risk for stroke and mortality [13], [14].

The major factors influencing serum creatinine are muscle mass and GFR. In this study, Hb-SS were comparable for age, weight, height and BMI with Hb-AA groups. In our series, creatinine tended to be lower in children with Hb-SS than in Hb-AA subjects (table-3). Similar findings were previously reported [15]–[18]. As children with Hb-SS have high GFR, the serum creatinine is lower than expected and is further lowered by the hypersecretion of creatinine. Consequently, this tendency was significantly decreased compared to corresponding values in Hb-AA group. Thus, interpretation of serum creatinine must be sensitive to this factor. Values at the upper end of the normal range should raise the index of suspicion for reduced renal function.

GFR was markedly increased in Hb-SS group compared to Hb-AA group. This observation appears in line with such evidence that increase of GFR is predominantly in sickle cell pediatric series [17]–[20]. In contrast, Glomerular filtration rate did not differ significantly between the age groups in both patients and normal controls in Nigerian series [21], [22]. We therefore speculate that genetic factors as difference of haplotype between Congolese and Nigerian children suffering from SCD may explain this difference. The association between CAR haplotypes and high risk of sickle cell nephropathy has been described in the literature [3], [8].

In this study, glomerular dysfunction in this population is found to be important. Hyperfiltration was present in 30.8% of children with Hb-SS. This is disturbing low when compared with prevalence of 76% reported by Aygun et *al* in USA [20]. The prevalence of hyperfiltration in our group was lower probably because of the method used for GFR estimation in this work was different than Tc 99 clearance used by Aygun in USA [20]. Hyperfiltration is common in young patients suffering from SCD due to glomerular hypertrophy [6]. More recently, this theory has been challenged and suggests that increased nitric oxide synthetase activity leads to glomerular hyperfiltration in sickle cell disease [23].

In SCD, there are several abnormalities in proximal tubular function with increased rate of creatinine secretion. In this condition, creatinine clearance overestimates the rate of GFR.

Renal insufficiency (<90 ml/min/m^2^) was present in 12.3% of children suffering from SCD. Prevalence of renal complications in our cohort was found to be not negligible. In USA, Sklar et *al* noted that 10% of patients in their series developed an increase in creatinine.

Persistent proteinuria is believed to be precursor of chronic renal failure in sickle cell patient. Ratio of albumin to creatinine (ACR) was not available in DRC. In our series, prevalence of proteinuria detected by dipstick was 6.2% consistent with the 2.8–6.2% reported in USA and Ghana [19], [24], [25]. One child with Hb-SS was found to have asymptomatic nephrotic proteinuria. Nephrotic syndrome in children suffering from SCD has been reported by African authors [26]–[28].

Non-availability of appropriate diagnostic tools remains a formidable hindrance to be surmounted in the establishment of prevalence of proteinuria in paediatric population suffering from SCD in resource-limited settings. The true prevalence of proteinuria was probably less than indicated since (i) an inability to concentrate urine normally is frequent in sickle cell patient. In this condition, reagent strip analysis probably underestimates the presence of proteinuria (ii) in this study, microalbuminuria was not done to confirm that proteinuria was present in children tested negative for proteinuria with a dipstick method. In previous studies, the prevalence of proteinuria significantly increases when microalbuminuria was assessed [29], [30].

In this study, uric acid levels were significantly higher in Hb-SS than in control. Our observation is similar to previous studies [31]. In SCD, uricemia is due to the increase in urate production associated with accelerated erythropoiesis [32].

Hyperuricemia was infrequent in children with sickle cell anemia in our series. Hyperuricemia was only found in 7.7% in Hb-SS group. This frequency is comparable with a previous report brought out by Thomspon et *al* who reported in 15% of children with SCD [33] but is lower than that reported by others studies which reported frequencies ranging from 28% to 75% of affected children with SCD [15], [34], [35]. This difference should be associated to factors influencing uric acid such as diet, genetic predisposition or differences in the assays of uric acid. No case of gout was reported in our series as reported by previous studies [15], [36].

In this study, urea tended to be significantly lower in children with Hb-SS than in Hb-AA. This observation is similar to previous reports [15], [16].

## Conclusions

Glomerular dysfunction is not uncommon and may be under-reported in children with sickle cell disease. In this study, it appears that one in 3 children had hyperfiltration. In a resource-limited setting, hyperfiltration is a major indicator of deterioration in renal function, which occurs earlier than decreased creatinine clearance and/or proteinuria.

Further investigation will be important to identify sickle cell children at risk of glomerular dysfunction and is warranted to elucidate the cause and to allow for earlier therapeutic intervention to decrease incidence and prevalence of sickle cell nephropathy in Congolese environment.
